# Comparison of Scarf With and Without Akin Osteotomy for Hallux Valgus

**DOI:** 10.7759/cureus.96498

**Published:** 2025-11-10

**Authors:** Adham M Abdulsamad, Abdulrahman H Alfarag, Ohud Alsaqer, Abdulrahman H Alqahtani, Abdulelah Y Alhawas, Talal K Alassaf, Turki N Alharbi, Mohammed A Alsaif, Sultan Alqasim, Mohammad H Albadr

**Affiliations:** 1 Department of Surgery, Division of Orthopedic Surgery, King Abdulaziz Medical City, Ministry of National Guard Health Affairs, Riyadh, SAU; 2 College of Medicine, King Saud Bin Abdulaziz University for Health Sciences, Riyadh, SAU

**Keywords:** akin osteotomy, bmi, hallux valgus, postoperative pain, radiological outcomes, scarf osteotomy

## Abstract

Introduction

Hallux valgus (HV) is a common foot deformity characterized by misalignment of the first metatarsophalangeal joint, often impacting quality of life. Its causes are multifactorial, including genetics, lifestyle, and improper footwear. Management ranges from conservative approaches to surgical correction, with Scarf osteotomy commonly used. This study aims to compare outcomes of Scarf osteotomy with and without Akin osteotomy in a Saudi population.

Methodology

This is a retrospective cohort study conducted at King Abdulaziz Medical City (KAMC), Riyadh, Saudi Arabia, analyzing outcomes of Scarf osteotomy with and without Akin for a total of 148 patients. The study included all patients who underwent the procedure between 2016 and 2024 who had available and accessible medical records. The surgeries were performed and supervised by an entire team consisting of three consultants with fellowship training in foot and ankle surgery in KAMC Riyadh. Data from 2016 to 2024 were extracted from BestCare (a health information system for medical records), using convenience sampling. The data were cleaned in Excel (Microsoft® Corp., Redmond, WA) and analyzed in Statistical Product and Service Solutions (SPSS, version 29.0.0; IBM SPSS Statistics for Windows, Armonk, NY).

Results

This study included 148 patients (177 feet) undergoing Scarf osteotomy (Scarf alone: 75 (50.7%), Scarf with Akin: 73 (49.3%)). All patients were followed for at least one year after surgery, providing enough time to observe long-term outcomes and recurrence. The majority were female (140 (94.6%)), aged 31-40 years (42 (28.4%)). Post-operative pain was reported in 49 (33.1%), recurrence in 12 (8.1%), and non-union in 6 (4.1 %). Radiologically, significant improvements were observed in the intermetatarsal angle (IMA) (right: -7.48°, p<0.001; left: -9.32°, p<0.001), HV angle (HVA) (right: -20.27°, p<0.001; left: -21.92°, p<0.001), and distal metatarsal articular angle (DMAA) (right: -10.53°, p<0.001; left: -12.00°, p<0.001). Scarf with Akin showed superior angular correction (HVA reduction: 22.92° vs. 17.50°; p<0.001). Among severe cases (n=31), the IMA improved by 10.37° vs 9.25° (right) and 11.92° vs 12.42° (left) in the Scarf with Akin and Scarf alone groups, respectively. HVA correction was greater with Scarf and Akin - 26.00° vs 23.00° (right) and 29.83° vs 28.86° (left) - demonstrating its superior efficacy. Postoperative pain occurred in 31.7% of patients, with higher rates in the Akin group.

Conclusion

This study shows both Scarf and Scarf with Akin osteotomies effectively corrected HV deformities with low complication rates. Scarf with Akin achieved greater angular correction, particularly in severe deformities, but at the cost of slightly increased postoperative pain. However, Scarf alone had slightly better sesamoid realignment. Postoperative pain was the most common complication, with BMI being the only significant predictor, highlighting the importance of weight management in surgical recovery.

## Introduction

Hallux valgus (HV) is a metatarsophalangeal (MTP) deformity defined by a complex valgus deformity of the first ray. The proximal phalanx is valgus deviated and pronated with concurrent varus deviation of the first metatarsal bone, leading to widened angulation of the first MTP joint [1]. Patients with HV usually have difficulty in finding appropriately fitting shoes because of the deformity. HV can greatly affect quality of life as it is associated with pain and severity, which are associated with the severity of deformity [2]. This condition is recognized as one of the most common foot disorders, with significant variations in prevalence across different populations and demographics. A systematic review and meta-analysis on the global prevalence and incidence of HV showed that the pooled prevalence of HV is notably higher in females (23.74%) compared to males (11.43%) [3]. A retrospective cohort study on 166 patients who underwent corrective osteotomy for HV at King Abdulaziz Medical City (KAMC) in Riyadh, Saudi Arabia, showed that the majority (91.6%) of the participants were female and the mean age was 41.3 years [4]. HV is relatively rare in younger populations, with a prevalence of 11% in individuals under 20 years old, increasing to 12.22% in adults aged 20-60 years, and reaching as high as 22.7% in those over 60 years old [3]. This trend is corroborated by another research, which shows that HV affects approximately 35.7% of older adults [5]. The progressive nature of the deformity, combined with the cumulative effects of lifestyle and footwear choices over time, likely contributes to this increased prevalence in older populations [6]. Causes tend to be multifactorial, and many risk factors have been illustrated in the literature, making HV the result of genetic predisposition, lifestyle factors, and chronic diseases, rather than a single known cause [1]. However, genetic predisposition in this condition plays a major role, with 70% of patients with HV having a positive family history. Other risk factors include pes planus, rheumatoid arthritis, and inappropriate shoe sizing [7]. Management of HV starts with a nonoperative method in all patients, and this mainly includes proper shoes with pads or orthoses. If nonoperative management fails, and the patient still complains of pain or difficulty walking, then surgery is indicated. Many surgical approaches exist, and all depend on the severity of the deformity and the overall status of the patient. Surgical options include osteotomy, arthrodesis, and arthroplasty, with osteotomy being the most commonly done since many patients present with mild deformities [8]. Scarf osteotomy is an option out of a variety of other osteotomies, and in some cases, they perform Akin osteotomy as augmented correction. Scarf osteotomy involves a Z-shaped cut in the metatarsal, allowing for translation and rotation to correct the alignment of the first metatarsal [9]. The addition of an Akin osteotomy, which targets the proximal phalanx, has been proposed as a complementary procedure to enhance the overall correction of the HV deformity, particularly in cases with significant interphalangeal deformity [10]. The Akin approach is usually reserved for more severe cases; when it is done in select cases, it shows a lower recurrence rate and improvement in pain [11]. Adding an Akin osteotomy has been shown in several studies to enhance radiographic correction, particularly in patients with moderate-to-severe deformities or when there is incomplete correction after metatarsal realignment alone. This combination may reduce the risk of recurrence and improve cosmetic outcomes, although it slightly increases surgical complexity. Therefore, understanding when to add Akin to the Scarf osteotomy is crucial for achieving optimal deformity correction and patient satisfaction [11,12]. Since this approach is now more liberal and is done for many patients in NGHA-KAMC, comparing the parameters in addition to infection rate and overcorrection rate is essential to decide whether to go with this approach or only go when indicated or with select cases. The main objective of this study is to compare the clinical and radiological outcomes of scarf osteotomy performed with and without Akin osteotomy for the correction of HV deformity in Saudi Arabia, since it is being done more frequently, but evidence is still not abundant.

## Materials and methods

A retrospective cohort study was conducted at KAMC in Riyadh, Saudi Arabia. The study compared the clinical and radiological outcomes of Scarf osteotomy performed with and without Akin osteotomy for the correction of HV deformity. The data were collected by the study authors through accessing patients' medical and surgical histories from the BestCare system (health information System for medical records).

All patients aged 18 and above who underwent Scarf osteotomy with or without Akin between January 2016 and October 2024 were included in the study. Patients under the age of 18, HV angle (HVA) less than 15 degrees, intermetatarsal angle (IMA) less than 9 degrees, patients who underwent additional surgery in the same foot, or patients with neuromuscular disease affecting foot mechanics were all excluded. Based on data of interest, the research team created a data collection sheet, which included patients’ age, gender, BMI, medical history, surgery type and date, angles pre and post OP, and postoperative complications.

Collected data were analyzed through Statistical Product and Service Solutions (SPSS, version 29.0.0; IBM SPSS Statistics for Windows, Armonk, NY). Percentages and frequencies were reported for categorical variables. Continuous variables were reported as mean and standard deviation (SD). The Pearson chi-square test was used to assess the association between categorical outcome variables. For continuous outcome variables, the paired sample t-test and repeated measure ANOVA were used to compare the means between two groups. A p-value of 0.05 or less was considered statistically significant.

## Results

Our study included 148 participants for comparing outcomes of Scarf osteotomy performed with and without Akin osteotomy for correction of HV deformity (Table 1). The minimum follow-up period for all patients in our study was one year, during which clinical outcomes and postoperative complications were monitored. Radiological assessment was based solely on the initial postoperative foot X-ray. Notably, the majority were female (n=140, 94.6%), with a small male representation (n=8, 5.4%). Most participants were aged between 31 and 40 years (n=42, 28.4%), followed by 18-30 years (n=32, 21.6%) and 41-50 years (n=30, 20.3%). The mean age was 42.5 years (SD=14.1). Regarding BMI, most were of normal weight (n=58, 39.2%), followed by obese (n=50, 33.8%) and overweight (n=34, 23.0%), with a mean BMI of 27.6 kg/m² (SD=6.6). Only a small proportion had diabetes (n=17, 11.5%). Surgical interventions were nearly evenly split between Scarf alone (n=75, 50.7%) and Scarf with Akin (n=73, 49.3%). Surgeries were most frequently performed on the right foot (n=34, 23.0%) and right foot with Akin (n=32, 21.6%), with bilateral procedures being less common (Scarf with Akin: n=15, 10.1%; Scarf alone: n=11, 7.4%).

**Table 1:** Sociodemographic and surgical parameters of participants (n=148) Data are presented as frequencies (N, %), mean ± standard deviation (SD), and range where appropriate.

Variables		Frequency N (%)
Gender	Female	140 (94.6%)
	Male	8 (5.4%)
Age (Years)	18–30 Years	32 (21.6%)
	31–40 Years	42 (28.4%)
	41–50 Years	30 (20.3%)
	51–60 Years	25 (16.9%)
	>60 Years	19 (12.8%)
	Mean (Sd)	42.5 (14.1)
	Range	18-79
BMI (kg/m^2^)	Underweight	6 (4.1%)
	Normal	58 (39.2%)
	Overweight	34 (23.0%)
	Obese	50 (33.8%)
	Mean (Sd)	27.6 (6.6)
	Range	16.4-49.5
Medical Hx (DM and PAD)	No	131 (88.5%)
	Yes (DM)	17 (11.5%)
Surgery Type	Scarf Alone	75 (50.7%)
	Scarf with Akin	73 (49.3%)
Surgery Type Based on Foot	Bilateral Scarf	11 (7.4%)
	Bilateral Scarf with Akin	15 (10.1%)
	Left Scarf	30 (20.3%)
	Left Scarf with Akin	26 (17.6%)
	Right Scarf	34 (23.0%)
	Right Scarf with Akin	32 (21.6%)

**Figure 1:**
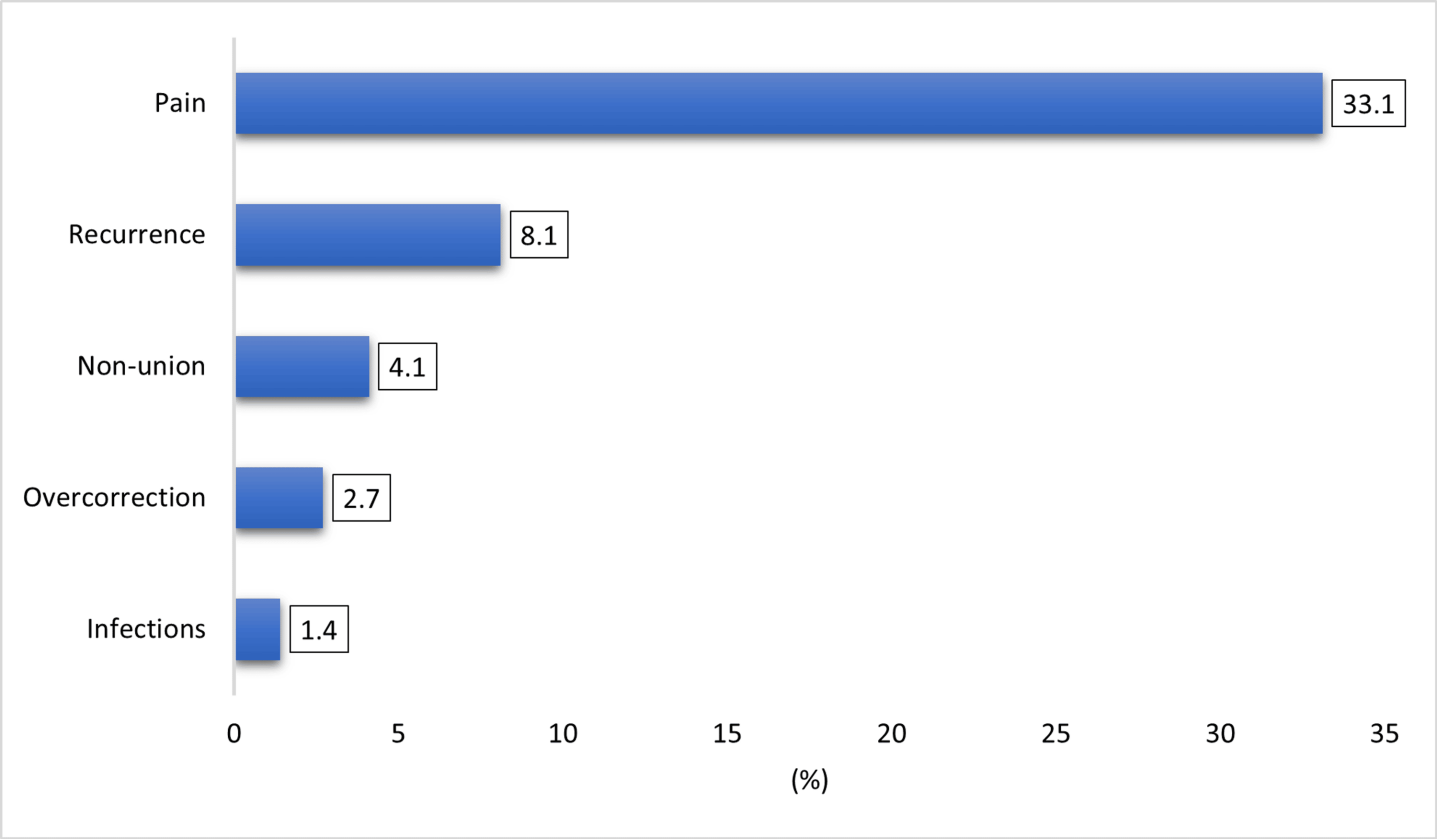
Postoperative clinical outcome in participants after Scarf osteotomy (n=148) Data are presented as percentages (%).

Figure 1 shows the primary postoperative clinical outcomes among participants following Scarf osteotomy. The most commonly reported complication was pain that was assessed from one month to one year post-op (n=49, 33.1%), followed by recurrence (n=12, 8.1%). Other observed complications included non-union (n=6, 4.1%), overcorrection (n=4, 2.7%), and infections (n=2, 1.4%).

Table 2 shows the postoperative radiological outcomes in participants (n=148) following Scarf osteotomy, with significant clinical improvements. On the right foot, the IMA decreased from 16.32 (SD=3.40) to 8.84 (SD=4.67), a mean reduction of 7.48 (p<0.001). HVA improved from 34.66 (SD=9.15) to 14.39 (SD=9.43), and distal metatarsal articular angle (DMAA) from 20.72 (SD=7.02) to 10.19 (SD=5.30), both statistically significant (p<0.001). Sesamoid alignment improved, with displaced cases dropping from 88 (59.5%) to 53 (35.8%), while aligned cases rose from 6 (4.1%) to 40 (27.0%) (p<0.001). On the left foot, similar trends were observed. IMA decreased from 17.77 (SD=3.83) to 8.45 (SD=4.04), and HVA from 36.83 (SD=10.42) to 14.91 (SD=9.65), both with p<0.001. DMAA also reduced significantly from 20.63 (SD=7.73) to 8.63 (SD=4.57). Sesamoid displacement decreased from 81 (54.7%) to 48 (32.4%), and alignment improved from 2 (1.4%) to 34 (23.0%) (p<0.001).

**Table 2:** Postoperative radiological outcome in participants after Scarf osteotomy (n=148) Data are presented as mean ± SD for continuous variables (IMA, HVA, DMAA) and as N (%) for categorical variables (sesamoid displacement/alignment). A p-value <0.05 was considered statistically significant. Paired sample t-test was used for continuous variables, and McNemar test for categorical variables. (a) Paired sample t-test, (b) McNemar test DMAA: distal metatarsal articular angle; HVA: hallux valgus angle; IMA: intermetatarsal angle

Variables			Pre-Op Mean (SD)	Post-Op Mean (SD)	Mean Difference	t/χ^2^ value	Sig. Value
Right Sided	IMA		16.32 (3.40)	8.84 (4.67)	-7.48	15.69	<0.001^a^
	HVA		34.66 (9.15)	14.39 (9.43)	-20.27	17.49	<0.001^ a^
	DMAA		20.72 (7.02)	10.19 (5.30)	-10.53	12.27	<0.001^ a^
	Sesamoid	Displaced	88 (59.5%)	53 (35.8%)	-23.7%	31.03	<0.001^ b^
		Aligned	6 (4.1%)	40 (27.0%)	+22.9%		
Left Sided	IMA		17.77 (3.83)	8.45 (4.04)	-9.32	24.73	<0.001^ a^
	HVA		36.83 (10.42)	14.91 (9.65)	-21.92	17.94	<0.001^ a^
	DMAA		20.63 (7.73)	8.63 (4.57)	-12.00	12.21	<0.001^ a^
	Sesamoid	Displaced	81 (54.7%)	48 (32.4%)	-22.3%	28.26	<0.001^ b^
		Aligned	2 (1.4%)	34 (23.0%)	+21.6%		

Table 3 shows the effectiveness of Scarf alone versus Scarf with Akin procedures on radiological outcomes for both right and left sides. On the right side, IMA improved from 15.52° (SD=3.05) to 8.52° (SD=4.17) in Scarf alone (Δ=-7.00°), while Scarf with Akin showed a larger correction from 17.08° (SD=3.57) to 9.15° (SD=5.13) (Δ=-7.93°, p<0.001). HVA was reduced by 17.50° in Scarf alone and 22.92° in Scarf with Akin. DMAA improved by 10.21° and 10.84°, respectively. Right sesamoid displacement dropped more in Scarf alone (-39.2%) than in Scarf with Akin (-34.2%). On the left, Scarf alone improved IMA by 8.85° and HVA by 19.25°, whereas Scarf with Akin achieved greater corrections of 9.76° and 24.46°, respectively. DMAA improved slightly more with Akin (Δ=12.36°). Left sesamoid displacement decreased more in Scarf alone (-45.1%) than in Akin (-33.3%). Both techniques yielded significant improvements (p<0.001), though Akin was slightly superior in angular correction.

**Table 3:** Comparison and effectiveness of different radiological outcomes between two operative procedures of Scarf osteotomy (n=148) Data are presented as mean ± SD for continuous variables (IMA, HVA, DMAA) and as N (%) for categorical variables (sesamoid displacement/alignment). A p-value <0.05 was considered statistically significant. Between-group comparisons were performed using repeated measures ANOVA. (a) Repeated measure ANOVA DMAA: distal metatarsal articular angle; HVA: hallux valgus angle; IMA: intermetatarsal angle

Variables			Scarf Alone			Scarf with Akin			F Value	Sig. Values
			Pre-Op Mean (SD)	Post-Op Mean (SD)	Mean Difference	Pre-Op Mean (SD)	Post-Op Mean (SD)	Mean Difference		
Right Side	IMA		15.52 (3.05)	8.52 (4.17)	7.00	17.08 (3.57)	9.15 (5.13)	7.93	1329.38	<0.001^a^
	HVA		33.39 (8.19)	15.89 (9.12)	17.50	35.88 (9.91)	12.96 (9.58)	22.92	1022.69	<0.001^ a^
	DMAA		19.78 (6.31)	9.57 (5.39)	10.21	21.63 (7.59)	10.79 (5.21)	10.84	1065.65	<0.001^ a^
	Sesamoid N (%)	Displaced	43 (93.5%)	25 (54.3%)	-39.2%	45 (93.8%)	28 (59.6%)	-34.2%	1467.84	<0.001^ a^
		Aligned	3 (6.5%)	21 (45.7%)	+39.2%	3 (6.3%)	19 (40.5%)	+34.2%		
Left Side	IMA		17.15 (4.00)	8.30 (4.23)	8.85	18.36 (3.61)	8.60 (3.90)	9.76	1578.22	<0.001^ a^
	HVA		35.75 (10.50)	16.50 (10.03)	19.25	37.86 (10.35)	13.40 (9.13)	24.46	772.56	<0.001^ a^
	DMAA		19.68 (7.95)	8.05 (4.29)	11.63	21.55 (7.51)	9.19 (4.80)	12.36	865.57	<0.001^ a^
	Sesamoid N (%)	Displaced	40 (97.6%)	21 (52.5%)	-45.1%	41 (97.6%)	27 (64.3%)	-33.3%	1782.60	<0.001^ a^
		Aligned	1 (2.4%)	19 (47.5%)	+44.1%	1 (2.4%)	15 (35.7%)	+33.3%		

Table 4 shows radiological outcomes among patients with severe HV (n=31) undergoing either Scarf alone or Scarf with Akin procedures. On the right side, Scarf alone corrected IMA by 9.25°, HVA by 23.00°, and DMAA by 14.50°, whereas Scarf with Akin achieved more corrections: IMA by 10.37°, HVA by 26.00°, and DMAA by 18.12° (p<0.001 for all). Sesamoid alignment improved more in Scarf alone (+18.2%) than in Akin (+10.0%). On the left side, IMA correction was slightly greater with Scarf alone (mean differences: 12.42°) compared to Scarf with Akin (mean differences: 11.92°), though HVA and DMAA showed more marked reductions in Scarf with Akin (mean differences: 29.83° and 13.00°, respectively). Interestingly, sesamoid alignment, which is defined as re-centered by about 5-10 mm beneath the first metatarsal head, improved more in Scarf alone (+27.3%) than with Akin (+10.0%).

**Table 4:** Comparison and effectiveness of different radiological outcomes between the two operative procedures of Scarf osteotomy among patients with patients with severe angles (n=31) Data are presented as mean ± SD for continuous variables (IMA, HVA, DMAA) and as N (%) for categorical variables (sesamoid displacement/alignment). A p-value <0.05 was considered statistically significant. Between-group comparisons were performed using repeated measures ANOVA. (a) Repeated measure ANOVA DMAA: distal metatarsal articular angle; HVA: hallux valgus angle; IMA: intermetatarsal angle

Variables			Scarf Alone			Scarf with Akin			F Value	Sig. Values
			Pre-Op Mean (SD)	Post-Op Mean (SD)	Mean Difference	Pre-Op Mean (SD)	Post-Op Mean (SD)	Mean Difference		
Right Side	IMA		19.50 (2.38)	10.25 (7.72)	9.25	20.75 (2.96)	10.38 (7.78)	10.37	93.79	<0.001^a^
	HVA		46.25 (2.63)	23.25 (13.60)	23.00	44.00 (5.78)	18.00 (13.44)	26.00	156.15	<0.001^ a^
	DMAA		22.00 (8.68)	7.50 (6.56)	14.50	28.00 (12.65)	9.88 (4.36)	18.12	98.42	<0.001^ a^
	Sesamoid N (%)	Displaced	4 (36.4%)	2 (18.2%)	-18.2%	8 (40.0%)	3 (15.0%)	-25.0%	356.43	<0.001^ a^
		Aligned	0 (0.0%)	2 (18.2%)	+18.2%	0 (0.0%)	2 (10.0%)	+10.0%		
Left Side	IMA		22.71 (4.19)	10.29 (4.86)	12.42	21.25 (2.05)	9.33 (4.58)	11.92	448.87	<0.001^ a^
	HVA		51.29 (10.95)	22.43 (7.72)	28.86	45.50 (4.01)	15.67 (9.76)	29.83	572.23	<0.001^ a^
	DMAA		26.43 (10.91)	9.14 (3.98)	17.29	24.17 (6.09)	11.17 (4.28)	13.00	245.93	<0.001^ a^
	Sesamoid N (%)	Displaced	7 (63.6%)	3 (27.3%)	-36.3%	12 (60.0%)	10 (50.0%)	-10.0%	1086.87	<0.001^ a^
		Aligned	0 (0.0%)	2 (27.3%)	+27.3%	0 (0.0%)	2 (10.0%)	+10.0%		

Table 5 compares outcomes between Scarf alone and Scarf with Akin procedures among participants. Among patients with comorbid diabetes, eight (47.1%) underwent Scarf alone, and nine (52.9%) had Scarf with Akin. Non-union was equally distributed with 3 (50.0%) cases in each group. Overcorrection occurred only in the Akin group (3, 100.0%), while none were reported in Scarf alone. Postoperative pain was reported in 23 (48.9%) Scarf alone patients and 24 (51.1%) Akin patients. Infection occurred exclusively in the Akin group (2, 100.0%), and not in the Scarf alone group. Recurrence was slightly more frequent in Scarf alone (8, 61.5%) than in Akin (5, 38.5%).

**Table 5:** Comparison of clinical outcomes between two operative procedures of Scarf osteotomy (n=148) Data are presented as N (%) for categorical variables. A p-value <0.05 was considered statistically significant. Between-group comparisons were performed using Chi-Square test or Fisher’s Exact test, as appropriate. (a) Chi-square test, (b) Fisher’s exact test

Variables	Answers	Surgery Type		χ^2^ value	Sig. values
		Scarf Alone N (%)	Scarf with Akin N (%)		
Comorbidity	No	67 (51.1%)	64 (48.9%)	0.101	0.751^ a^
	Yes (DM)	8 (47.1%)	9 (52.9%)		
Non-Union	No	72 (50.7%)	70 (49.3%)	0.001	1.000 ^b^
	Yes	3 (50.0%)	3 (50.0%)		
Overcorrection	No	75 (51.7%)	70 (48.3%)	3.146	0.117^ b^
	Yes	0 (0.0%)	3 (100.0%)		
Pain	No	52 (51.5%)	49 (48.5%)	0.038	0.773^ a^
	Yes	23 (48.9%)	24 (51.1%)		
Infections	No	75 (51.4%)	71 (48.6%)	2.083	0.242^ b^
	Yes	0 (0.0%)	2 (100.0%)		
Recurrence	No	67 (49.6%)	68 (50.4%)	0.673	0.412^ a^
	Yes	8 (61.5%)	5 (38.5%)		

Table 6 compares clinical outcomes in patients with severe HV (n=31) treated with either Scarf alone or Scarf combined with Akin osteotomy. Although no statistically significant differences were observed between the two groups (p > 0.05), some variations were noted. Diabetes occurred equally across both groups (50% for each group). However, 68.0% of patients in the Scarf with Akin group had no additional comorbidities, compared to 32.0% in the Scarf alone group. Postoperative pain was more common in the Akin group (72.7%) than in the Scarf alone group (27.3%). Recurrence and non-union rates were similar in both groups, and no cases of overcorrection or infection were reported.

**Table 6:** Comparison of clinical outcomes between two operative procedures of Scarf osteotomy (n=31) Data are presented as N (%) for categorical variables. A p-value <0.05 was considered statistically significant. Between-group comparisons were performed using the chi-square test or Fisher’s exact test, as appropriate. (a) Chi-square test, (b) Fisher’s exact test

Complication		Surgery Type		χ^2^ value	Sig. values
		Scarf Alone N (%)	Scarf with Akin N (%)		
Comorbidity	No	8 (32.0%)	17 (68.0%)	0.685	0.638^ b^
	Yes (DM)	3 (50.0%)	3 (50.0%)		
Non-Union	No	9 (33.3%)	18 (66.7%)	0.423	0.601^b^
	Yes	2 (50.0%)	2 (50.0%)		
Pain	No	8 (40.0%)	12 (60.0%)	0.502	0698^ b^
	Yes	3 (27.3%)	8 (72.7%)		
Recurrence	No	9 (34.6%)	17 (65.4%)	0.053	1.000^ b^
	Yes	2 (40.0%)	3 (60.0%)		

Table 7 shows the adjusted predictors of pain as a poor outcome after Scarf osteotomy using logistic regression analysis. Among the variables, BMI was the only significant predictor of postoperative pain (B = 0.106; p = 0.001), indicating that each unit increase in BMI raised the odds of pain by 11.2% (OR = 1.112, 95% CI: 1.044-1.184). Other variables - gender (OR = 1.710, p = 0.498), age (OR = 0.980, p = 0.223), and comorbidity with diabetes (OR = 1.496, p = 0.533) - were not statistically significant predictors.

**Table 7:** Adjusted predictors of pain as poor outcome after Scarf osteotomy (logistic regression analysis) Data are presented as regression coefficients (B), odds ratios (Exp(B)), and 95% confidence intervals (CI). A p-value <0.05 was considered statistically significant. Logistic regression analysis was used to identify predictors of post-operative pain. B: coefficient of the regression model, Exp(B): Odds ratio, CI: confidence interval

Variables	B	Sig.	Exp(B)	95% CI	
				Lower	Upper
Gender	0.536	0.498	1.710	0.363	8.053
Age	-0.020	0.223	0.980	0.948	1.012
BMI	0.106	0.001	1.112	1.044	1.184
Comorbidity (DM)	0.403	0.533	1.496	0.421	5.314
Constant	-2.959	0.001	0.052		

## Discussion

HV is a complex forefoot deformity, which is commonly managed by various surgical techniques aiming to correct angular misalignment, restore normal joint congruence, and reduce pain [13]. Our study aimed to compare the clinical and radiological outcomes of Scarf osteotomy performed with and without the Akin procedure among participants with mild-to-moderate and severe angle deformity. According to the study by Ray et al., the severe angles are defined as HVA > 40 IMA > 16 [14].

There is an important finding of this investigation that the overall success of Scarf osteotomy is reflected by the marked radiological improvements for both the right and left sides. In line with this classification, our study demonstrated that all three key radiological angles: IMA, HVA, and DMAA decreased significantly from preoperative to postoperative measurements, indicating that Scarf osteotomy effectively corrected the angular deformities even in severe cases. Similar improvements have been documented in previous studies evaluating Scarf osteotomy, where the procedure reliably corrects angular deformities, alleviates pain, and enhances foot function [15]. Our results align with those of Butler et al. [11] and Kaufmann et al. [12], who found a substantial reduction in both HVA and IMA following Scarf steotomy, underscoring its efficacy as a midshaft procedure with intrinsic stability that allows early weight-bearing [12,13].

When evaluating the outcomes of Scarf alone versus Scarf with Akin, both in mild-to-moderate and severe HVA, we observed that both approaches significantly improved radiological parameters (p<0.001). Moreover, if we add an Akin osteotomy, it demonstrates slightly superior angular corrections in certain metrics, such as the mean reduction in HVA and IMA. This is consistent with prior literature suggesting that the Akin procedure can further optimize correction, particularly in cases where there is a residual hallux interphalangeal component or when the proximal phalanx alignment also needs addressing [16]. Similarly, according to a study by Butler et al. [11], if a Scarf-Akin approach is used, it yields a more accurate deformity correction in patients with moderate-to-severe HV angles; thus, an additional phalangeal osteotomy refines alignment [11]. Moreover, in our data, we observe a minimal difference, which may reflect that some patients had only mild-to-moderate angles or that surgeons selectively applied Akin based on intraoperative decision-making to achieve better cosmetic and functional outcomes.

Notably, the rates of key complications for all cases, such as non-union, recurrence, overcorrection, and infection, were generally low in both groups. Overcorrection appeared in the Scarf with Akin cohort (three patients, 100.0%), but not in the Scarf alone group. While this discrepancy was not statistically significant, it echoes findings from certain reports that highlight a slightly higher risk of overcorrection when additional osteotomies are performed, likely due to the inherent complexity of the procedure [17]. Despite that, the overarching outcome profile in both groups remained positive, indicating that, with appropriate surgical technique and patient selection, the risk of overcorrection and other complications can be minimized.

One of the most common complications in our participants was pain, which occurs after surgery and affects 33.1% (n=49) of participants. These surgeries, such as Scarf and Akin osteotomy for HV, realign and stabilize the first metatarsal that causes initial discomfort and persistent pain. The logistic regression analysis in our study identified body mass index (BMI) as a significant predictor for persistent postoperative pain. According to logistic regression analysis, each unit increase in BMI increases the chance of pain by approximately 11.2% in our participants. This finding is consistent with broader orthopedic literature, which suggests that higher BMI negatively affects surgical outcomes, prolongs healing times, and puts additional mechanical stress on the forefoot [18]. Karanth et al. [19] also show that obese patients tend to report increased pain scores postoperatively due to biomechanical loadings; thus, weight management is important in optimizing surgical results and patient satisfaction [19].

However, gender, age, and diabetes mellitus did not emerge as significant predictors of pain in our cohort. Although some previous studies (e.g., Ahmed et al. [20]) highlight age and diabetic status as factors influencing overall healing and susceptibility to complications, our data did not corroborate such associations. The relatively low incidence of diabetes (11.5%) and small number of male participants (5.4%) could partly explain why these variables did not reach statistical significance. Additionally, improvements in perioperative medical management and modern surgical techniques might mitigate the potential risks posed by these factors.

Thus, the clinical outcomes underscore that Scarf osteotomy, whether performed alone or in conjunction with the Akin procedure, provides substantial radiological correction with low complication rates. Surgeons may prefer adding an Akin osteotomy when the phalangeal component of the deformity warrants additional correction, particularly for more severe presentations. Although the overall complication rates were low, the Scarf with Akin group showed numerically higher overcorrection (3/73= 4.1%) and infection (2/73= 2.7%) than Scarf alone (0/73= 0% for both). However, these differences were statistically insignificant (p = 0.117 and p = 0.242, respectively). Therefore, careful patient selection, precise preoperative planning, and meticulous intraoperative techniques remain essential.

Limitations and future directions

There are several limitations of this study, which include its retrospective design, relatively short follow-up duration, and lack of long-term functional outcome assessments. Additionally, the small sample size of male participants and limited diversity in comorbidity profiles may constrain generalizability. This is why future research should incorporate prospective designs and extend the follow-up period. These studies should stratify patients by HV severity and evaluate long-term patient-reported outcomes. Emphasis should be placed on multi-center studies, and objective gait analyses would further refine our understanding of optimal surgical techniques for HV correction across diverse populations.

## Conclusions

These findings confirm that Scarf osteotomy, performed with or without the Akin procedure, significantly improves radiographic angles and yields favorable clinical outcomes. While Scarf with Akin may offer marginally better correction in specific metrics, both procedures demonstrate comparable safety profiles overall. Higher BMI emerged as a significant predictor of persistent pain, underscoring the importance of weight management in achieving optimal postoperative results. Collectively, these results align with previous reports in the orthopedic literature and further clarify the nuanced role of adjunctive Akin osteotomy in refining correction for HV deformities.
